# TopoRF-Net: Topology-Aware Road Segmentation in Multi-Resolution Remote Sensing via Multi-Receptive Field Adaptation

**DOI:** 10.3390/s25247428

**Published:** 2025-12-06

**Authors:** Junjie Fu, Chenliang Wang, Hongchen Lv, Hao Lu, Wenjiao Shi, Xuefeng Liao

**Affiliations:** 1 SuperMap Software Co., Ltd., Beijing 100015, China; fu0834@gmail.com (J.F.); lvhongchen@supermap.com (H.L.); luhao@supermap.com (H.L.); 2 School of Data Science and Artificial Intelligence, Wenzhou University of Technology, Wenzhou 325000, China; wangcl@lreis.ac.cn; 3 Institute of Geographic Sciences and Natural Resources Research, Chinese Academy of Sciences, Beijing 100101, China; shiwj@lreis.ac.cn

**Keywords:** remote sensing imagery, semantic segmentation, road extraction, multi-receptive field, structural connectivity, topology-aware loss

## Abstract

In multi-resolution remote sensing imagery, roads typically exhibit sparse, elongated, and structurally complex morphological characteristics, posing formidable connectivity modeling challenges for semantic segmentation models. Existing approaches predominantly focus on pixel-level accuracy, often neglecting the topological integrity of road networks, which leads to frequent discontinuities and omissions in predicted results. To address this, this paper proposes an end-to-end road extraction framework equipped with multi-receptive field modeling and structural connectivity preservation capabilities. The model incorporates a multi-receptive-field module to capture road patterns across varying spatial scales, a connectivity-aware decoding mechanism to strengthen structural coherence, and a topology-aware loss that explicitly guides the restoration of continuous road networks during training. On the DeepGlobe-Road dataset, TopoRF-Net achieves OA 98.57%, IoU 69.76%, F1-score 82.18%, Precision 85.50%, and Recall 79.12%; on the Massachusetts dataset, TopoRF-Net similarly achieved outstanding results: OA 96.65%, IoU 59.68%, F1-score 74.75%, Precision 77.98%, and Recall 71.77%. These results conclusively demonstrate that the proposed method significantly outperforms existing approaches in both precision and connectivity metrics, whilst exhibiting favorable parameter efficiency and inference performance.

## 1. Introduction

**Figure 1:**
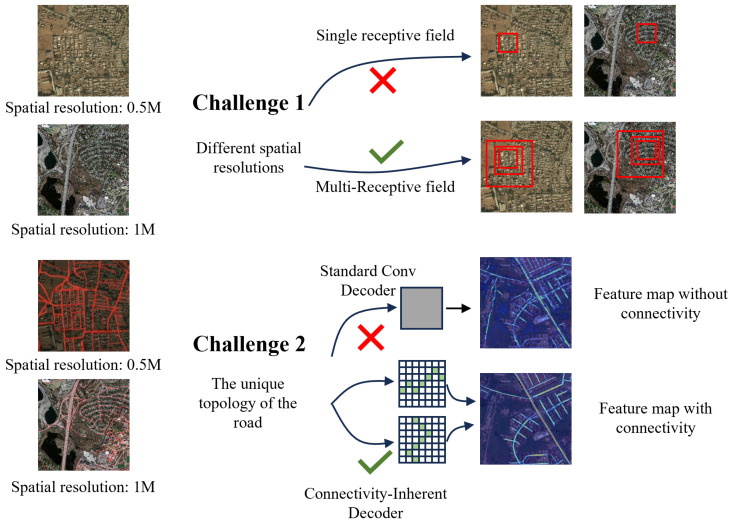
Two Major Challenges in Road Extraction from Remote Sensing Imagery.

Road extraction, as a critical task in remote sensing semantic segmentation, holds significant importance for urban planning, disaster response, and navigation systems [1,2,3]. However, unlike structured objects in natural imagery, roads in remote sensing images typically exhibit sparse distribution, elongated structures, and complex topological configurations. As shown in Figure 1, particularly in multi-resolution scenarios, severe mismatches in the receptive fields between images of different scales pose significant challenges for existing models in preserving road connectivity.

Most existing methods focus on pixel-level classification accuracy. They enhance contextual modeling with advanced encoder architectures such as Transformers [4,5], or pyramid-based multi-scale fusion modules [6,7]. While effective at semantic segmentation, these approaches often fail to maintain the global topological consistency of road networks, as they rely primarily on pixel-wise supervision and lack explicit constraints on geometric continuity and connectivity. This pixel-level optimization focuses mainly on local semantics. However, it overlooks the hierarchical nature of road networks. Large, coarse-scale structures determine the global layout, while small, fine-scale branches support local continuity. Ignoring this hierarchy often leads to fragmented or inconsistent road predictions. However, it overlooks the hierarchical nature of road networks. Large, coarse-scale structures determine the global layout, while small, fine-scale branches support local continuity. Ignoring this hierarchy often leads to fragmented or inconsistent road predictions. As a result, predictions frequently contain discontinuities, structural breaks, and omissions [8,9]. Vulnerable road segments such as narrow winding lanes and occluded areas are also prone to being missed, which further compromises network connectivity [1]. To overcome these issues, models require both cross-scale perception and fine-grained sensitivity to global path structures.

To address these challenges, we present Topology-Aware Multi-Receptive Field Network (TopoRF-Net), a connectivity-preserving framework for road extraction in multi-resolution remote sensing imagery. This framework is constructed based on CNNs and transformers, proposing a lightweight Multi-Receptive Field Enhancement (MRFE) module combined with a Connectivity-Inherent Decoder (CI-Decoder). This module adds parallel 3 × 3, 5 × 5, and 7 × 7 depth-separable convolution branches after each Transformer block, enabling robust multi-scale context modeling with minimal parameters and computation. For decoding, we propose a CI-Decoder, combining locally connected feature-aware convolutions with global feature aggregation to preserve road structural continuity. We also design a Connectivity-Constrained Training Strategy, which incorporates a topological consistency loss alongside Dice and cross-entropy losses. This loss encourages the model to prioritize the restoration of main road structures during training.

We evaluate TopoRF-Net on two representative datasets, DeepGlobe-Road and Massachusetts. The results demonstrate clear advantages in both accuracy and structural connectivity, while keeping the parameter size and inference cost competitive.

## 2. Related Work

### 2.1. Multi-Resolution Remote Sensing Scene Analysis

Multi-resolution remote sensing scenes present complex visual characteristics due to varying spatial resolutions, imaging conditions, and sensor modalities. These differences lead to significant variations in object appearance, scale, and texture across images. In particular, roads exhibit sparse distribution, elongated geometry, and intricate topological structures, making them especially challenging to identify and maintain connectivity across scales. These unique challenges become even more pronounced in multi-resolution scenarios, where significant mismatches in receptive fields between images of varying scales complicate road connectivity preservation.

In the field of remote sensing interpretation, deep learning has become an indispensable interpretative method. For semantic segmentation tasks, methods based on convolutional neural networks (CNNs) have established themselves as the mainstream approach due to their proficiency in capturing local features through convolution operations [10]. CNNs excel at learning local geometric patterns because each convolution kernel processes information within a limited spatial neighborhood, enabling the network to capture edges, textures, and other fine-grained structures that are spatially contiguous. The contextual understanding capabilities of these models are enhanced through multi-scale fusion approaches based on pyramids [6,7]. Recent research has further explored advanced CNN-based architectures to improve feature discrimination in complex remote sensing environments. For instance, a front–back view fusion (FBV-Fusion) strategy compatible with the YOLO framework was proposed to improve the detection of super tiny objects in high-resolution imagery, achieving superior performance across multiple datasets [11]. These methods excel at modelling the complex textures and patterns inherent in remote sensing data. However, convolutional neural networks are constrained by their finite receptive fields, hindering their ability to capture long-range dependencies. This poses a significant limitation when interpreting contextual information in large-scale scenes [12]. To overcome this shortcoming, researchers introduced the Transformer architecture to the visual domain [13]. Originally developed for natural language processing (NLP), Transformers gained prominence for their superiority in handling contextual relationships through self-attention mechanisms [14]. When adapted for visual tasks, Vision Transformers (ViTs) demonstrated outstanding performance in semantic segmentation [4,5,15], Their self-attention mechanism enables the modeling of long-range dependencies, providing stronger global context understanding. However, their computational complexity scales quadratically with sequence length, posing substantial challenges for processing high-resolution remote sensing imagery.

In response to these limitations, a number of studies have explored multi-scale feature fusion [16], efficient Transformer models [17], and context modeling techniques [18] to better handle cross-resolution challenges in remote sensing images. This is consistent with some research that effectively modeling both short- and long-range context is crucial for improving segmentation performance [19]. Such dual-context modeling helps to better capture the details of road structures while maintaining the overall coherence and continuity of the road network, which is particularly important for dealing with the topological complexities in road extraction.

### 2.2. Road and Line Feature Extraction

Road extraction constitutes a pivotal task in semantic segmentation for remote sensing applications [1,2,3]. Being linear structures, roads present challenges during identification, including discontinuities, occlusions, and shadows. Traditional approaches typically employ heuristic algorithms based on prior knowledge (e.g., edge detection and watershed segmentation), yet suffer from poor generalization. Contemporary methods predominantly utilize deep convolutional networks such as UNet [20] and other variants [21], aiming to decode local spatial context at varying levels of complexity [22,23], focus on specific contour features [24,25], or integrate all relevant features within the architecture [26,27,28,29]. The DeepLab series models [6] and HRNet model [30] achieved significant improvements in pixel-level accuracy. To enhance road continuity, some studies introduced graph models [9] or path-guided mechanisms [8], but these approaches suffer from high structural complexity and low inference efficiency. This approach integrates curve-aware processing with global fusion strategies, enhancing road structural integrity while maintaining lightweight performance. Yang et al. [31] constructed a convolutional neural network (CNN) employing a full-scale feature fusion scheme. This model incorporates conditional dilated convolutional modules to better utilize and aggregate semantic features. To better preserve detail within extracted road segments, Tan et al. [32] proposed a scale-sensitive network architecture that integrates a scale-sensitive module and a scale-fusion module for collaborative feature optimization. Zhu et al. [33] developed a global context-aware network model leveraging spatial contextual attributes and relationships to enhance semantic saliency in road regions. In the work of Wu et al. [34], multi-scale spatial contextual properties were investigated through a global spatial feature pyramid pooling strategy based on dilated convolutions. In contrast, Zhou et al. [35] proposed an edge- and topology-preserving network architecture to precisely delineate road contours while maintaining connectivity. Wu et al. [36] designed a Bi-HRNet architecture to simultaneously predict node heatmaps and directional connectivity, thereby enhancing the completeness and detail of road segmentation. To enhance feature embedding quality, Guan et al. [37] constructed a Feature Pyramid Network (FPN) architecture based on capsule primitives to explore more distinctive feature encoding. Wang et al. [38] stacked an encoder-decoder model enabling efficient feature propagation through internal convolutions, employing conditional random fields for post-processing to preserve road connectivity. Abdollahi et al. [39] proposed a residual U-Net architecture integrating shape and boundary learning principles to reduce discontinuities in road extraction. In contrast, Zao and Shi [40] developed a Richer U-Net model employing a detail recovery structure to mitigate omissions and commission errors, alongside defining an edge-focused loss function to enhance boundary details. Explorations have also recently been conducted based on the Mamba network architecture [41], which leverages a state space model for long-range spatial dependency modeling in road extraction tasks. In parallel, several studies have enhanced feature representation and connectivity preservation through diverse architectural innovations. For instance, Fan et al. [42] proposed BD-WNet, a boundary-decoupling W-shape network that strengthens edge awareness for optical road segmentation. Liu Huajun et al. [43] introduced an Adaptive Fourier Convolution Network for frequency-domain road segmentation, while Liu Guoqi et al. [44] developed a Residual Complex Fourier approach to enhance high-frequency structural representation. Tong et al. [45] developed a multiscale fusion attention network (MSFANet) to integrate spectral and spatial information across multi-spectral images. Zhang R. et al. [46] proposed D-FusionNet based on dilated convolutional blocks, while Zhang H. et al. [47] designed a Swin-GAT dual-stream hybrid network for high-resolution road extraction. In addition, Feng et al. [48] adapted the Segment Anything Model (SAM) for large-scale road extraction, demonstrating strong generalization in very-high-resolution imagery.

Beyond purely convolutional or transformer-based designs, graph-inspired and topology-guided frameworks have gained attention. Zao et al. [49] and Gu et al. [50] introduced topological and self-consistency constraints to improve structural continuity and reduce disconnection artifacts. Furthermore, Yan et al. [51] combined convolutional neural networks (CNNs) with graph neural networks (GNNs) to enhance semantic representation, where CNNs focused on feature extraction and GNNs [52,53] handled structural reasoning and route graph reconstruction.

### 2.3. Topological Structure Preservation and Loss Function Design

Traditional loss functions such as Cross-Entropy (CE) and Dice Loss have been widely adopted for pixel-level classification tasks. While these loss functions excel in optimizing point-wise classification accuracy, they are limited in their ability to preserve global structural continuity, especially in tasks involving elongated or topologically complex objects like roads. As such, retaining structural consistency across the entire object becomes challenging, leading to common issues like broken or incomplete structures in predicted outputs.

To address this limitation, significant advances have been made by introducing structure-aware network designs and loss functions aimed at preserving topological properties. Early works proposed modifications to conventional architectures, incorporating topological awareness directly into the network’s design. For instance, Batra et al. [9] introduced a novel approach where road connectivity is modeled as part of the segmentation task, thereby improving the connectivity and continuity of road networks. Their method jointly optimized segmentation and topology in an integrated learning framework, which resulted in more robust extraction of complex road structures from satellite imagery. Mosinska et al. [8] addressed the prevalent issue of topological errors in “automatic curve structure sketching,” pointing out that pixel-level losses (such as BCE) only perform local discrimination and cannot penalize significant topological disruptions caused by minute pixel errors. Methods based on loss functions [54,55,56] introduce metric-based methods during training to enforce additional constraints. These methods enhance the strong constraints for tubular structure segmentation. Reference [54] proposed a similarity measure called centerline Dice, which is computed at the intersection points between the segmentation mask and the skeleton. Reference [55] introduced a geometry-aware tubular structure segmentation method, the Deep Distance Transform (DDT). This method combines the intuition of skeletonization and the classic distance transform used for tubular structure segmentation. These approaches focus on maintaining the continuity of tubular structure segmentation. However, inaccuracies and offsets in the skeleton can affect the precision of the constraints. Reference [56] proposed a similarity metric to capture the topological consistency of the predicted segmentation and designed a loss function based on morphological closing operators for tubular structure segmentation. In [57], topological data analysis methods were combined with geometric deep learning models for fine-grained segmentation of 3D objects. These methods aim to capture the features of topological objects.

Moreover, the emergence of Topology-Aware Networks has been a key milestone in incorporating explicit topological considerations into the architecture itself. For example, Topoal [58] is a road network extraction model that uses topology-aware representation learning. This model introduces dedicated modules to preserve the structure of road networks during segmentation tasks, ensuring that spatial continuity is maintained in challenging scenarios such as varying resolutions or facing occlusions. These works highlight the importance of topological structure preservation, making a compelling case for more specialized architecture designs that explicitly focus on maintaining structural integrity during learning.

In this work, inspired by these studies, we define road topology features explicitly during the training phase. By introducing a Topology Loss in combination with traditional losses such as Dice and Cross-Entropy, we jointly optimize for both pixel-level accuracy and structural consistency. This approach helps the model focus on global connectivity during optimization, ensuring that the predicted roads are not only accurate at the pixel level but also maintain their structural integrity across the entire network.

By integrating topology-preserving losses into the network, we enhance the model’s ability to restore global road structures and fix fragmented or missing road sections, which is critical in practical applications such as urban planning, disaster response, and autonomous driving.

## 3. Methodology

This section provides a detailed introduction to the proposed TopoRF-Net framework, including an overview of its overall architecture, the core multi-receptive field feature augmentation encoder, the connectivity-inclusive decoder (CI-Decoder), and the training loss strategy.

### 3.1. Overall Architecture

**Figure 2:**
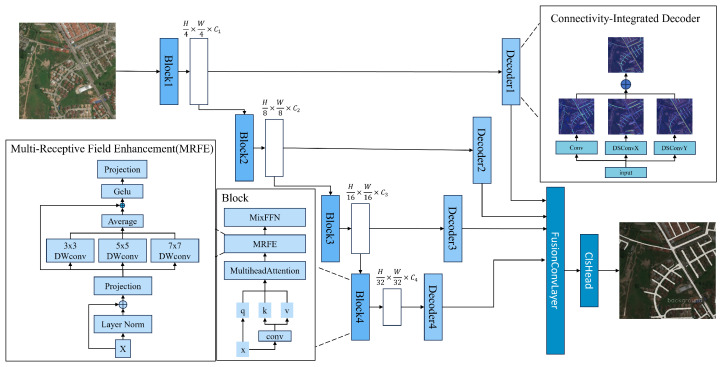
TopoRF-Net Structure Diagram: The encoder integrates a multi-scale MiT backbone with an MRFE module to enhance the receptive field, while the decoder incorporates a dynamic serpentine convolution structure to preserve road connectivity.

As shown in Figure 2, RoadNet-MS is constructed based on the MiT backbone. The overall architecture consists of four main modules:

In terms of overall framework design, this paper comprises three key components. First, during the encoder stage, we enhance the structure based on the MiT backbone network by inserting MRFE into each Transformer Block, as shown on the left side of Figure 2. The MRFE module comprises multi-head attention, a depthwise separable convolution group, and a residual path. This configuration enables effective modeling of fine-grained structures across different receptive fields, thereby enhancing the encoder’s sensitivity to elongated road features (see Section 3.2 for details).

Second, for the decoder stage, we propose the CI-Decoder, as shown on the right side of the figure. This decoder comprises three directional dynamic snake-shaped convolution modules (DSConv-X, DSConv-Y, and Conv-XY) alongside standard convolutional layers. It explicitly perceives geometric features of road curves and intersections, fuses features under a global gating mechanism, and ultimately outputs high-resolution semantic prediction maps (see Section 3.3 for details).

Finally, we introduce a topology-aware training mechanism. By jointly optimizing Dice loss, cross-entropy loss, and topology-preserving losses (e.g., Soft-Skeleton Loss), we reinforce the model’s capability in restoring main road structures and repairing fragmented regions. This constrains the model to focus on overall topological consistency from the early training stages (see Section 3.4 for details).

### 3.2. Multi-Receptive Field Enhancement Modules (MRFE)

To enhance the encoder’s perception of road structures at different scales, this paper proposes the MRFE, whose structure is shown on the left side of Figure 2. Embedded within each block of the MiT encoder, this module serves as an effective supplement to the Transformer’s self-attention mechanism. It expands the model’s spatial receptive field while maintaining a lightweight overall architecture, improving its ability to model winding and fine-grained structures.

Specifically, the input features of MRFE are denoted as $X\in R^{H\times W\times C}$, where *H* and *W* represent the spatial height and width of the feature map, and *C* denotes the number of channels. They first undergo layer normalization followed by weighted residual fusion with learnable scaling factors $s_{1}$ and $s_{2}$, and the operator LN(·) refers to *Layer Normalization*, which normalizes the feature values along the channel dimension. resulting in a normalized representation:

(1) $$X_{\mathrm{norm}}=s_{1}\cdot \mathrm{LN}\left(X\right)+s_{2}\cdot X$$

Subsequently, $X_{\mathrm{norm}}$ is fed simultaneously into three parallel depthwise separable convolution branches, with kernel sizes of 3×3, 5×5, and 7×7 respectively, to explicitly model local structures under different receptive fields:

(2) $$F_{k}={\mathrm{DWConv}}_{k}\left(X_{\mathrm{norm}}\right),\;k\in \left\{3,5,7\right\}$$

The feature maps from the three branches are subsequently aggregated into a single path through an averaging fusion operation:

(3) $$F_{\mathrm{avg}}=\frac{1}{3}\left(F_{3}+F_{5}+F_{7}\right)$$

The fused features undergo pointwise dimensionality projection and GeLU activation, enhancing nonlinear capabilities to generate the final output features:

(4) $$F_{\mathrm{out}}=\mathrm{GELU}\left(W_{\mathrm{up}}\cdot F_{\mathrm{avg}}\right)$$

Finally, the output features are concatenated with the original input *x* to form the module’s final output:

(5) $$X_{\mathrm{out}}=X+F_{\mathrm{out}}$$

The MRFE module offers three distinct advantages. First, its parallel multi-scale separable convolutional kernels capture both local and global semantic context without requiring a pyramid structure, significantly enhancing receptive field modeling capabilities. Second, thanks to its DW convolution architecture, the module remains extremely lightweight in terms of parameters and computational complexity. Finally, since the module does not rely on positional encoding, its inherent scale modeling capability naturally compensates for the spatial perception limitations of Transformers.

It is worth emphasizing that the MiT backbone network inherently possesses a four-stage multi-scale feature extraction mechanism, progressively downsampling from high resolution (Stage 1) to low resolution (Stage 4) to achieve semantic abstraction and contextual aggregation. The proposed MRFE module, embedded within each stage’s block, is inherently compatible with this hierarchical structure. This enables semantic features at every scale to possess rich multi-receptive field representation capabilities. Lower-level MRFE modules aid in capturing fine-grained structures like narrow roads and edges, while higher-level modules enhance the model’s perception of main roads, directionality, and global paths.

In summary, the enhanced encoder architecture proposed in this paper not only integrates the Transformer’s strengths in semantic modeling but also introduces the powerful expressive capabilities of convolutional structures for spatial modeling. This creates a unified, efficient feature extraction mechanism with outstanding structural awareness. Without significantly increasing model parameters or computational cost, this module substantially enhances the model’s responsiveness to slender, winding, and weakly textured road structures while providing high-quality multi-scale contextual information for subsequent topology-preserving decoders.

### 3.3. Connectivity-Inherent Decoder(CI-Decoder)

Road structures in remote sensing imagery often exhibit elongated, curved, and irregular spatial patterns, particularly in urban areas or under natural obstructions where numerous winding narrow roads or intersections exist. Traditional convolution operations perform fixed sampling on regular grids, making it difficult to effectively capture local directional changes and curvature trends. This leads to discontinuities, blurring, and structural misalignment in predicted road configurations.

This paper introduces a Dynamic Serpentine Convolution (DSC) [59] mechanism to enhance the model’s ability to capture local road curvature. The DSC module is embedded into each layer of the decoder, constructing direction-aware convolutional branches along both the lateral (X-axis) and longitudinal (Y-axis) dimensions to achieve two-dimensional curvature modeling.

In the initial stage, the DSC serves as a curvature-aware operator that adapts the sampling positions of the convolutional kernel to align with the local curvature of road structures. This mechanism allows the decoder to sensitively capture fine-grained bending patterns and irregular edges in individual directions. However, curvature perception alone primarily focuses on localized geometry and is insufficient for modeling the long-range structural continuity of entire road networks. To extend DSC from a purely curvature-aware module to a topology-aware representation, we decompose the serpentine convolution into two orthogonal branches, DSConv_*x*_ and DSConv_*y*_. These branches independently model lateral and longitudinal connectivity by tracing directional curvatures along the X and Y axes, respectively. Through joint fusion (Equation (9)), the decoder aggregates directional connectivity cues into a unified topological representation that captures both local curvature and global continuity. In this way, the transition from curvature-aware modeling (via DSC) to topology-aware learning (via the integration of DSConv_*x*_ and DSConv_*y*_) is achieved by embedding multi-directional connectivity constraints within the decoder. The resulting topology-enhanced feature representation enables consistent road reconstruction across occlusions, intersections, and discontinuities, ensuring robust structural completeness in the final segmentation map.

Specifically, assuming the input feature map is $X\in R^{H\times W\times C}$, snake convolution can be formalized as:

(6) $$y\left(p\right)=\sum_{k\in R}w_{k}\cdot X\left(p+\Delta_{k}\left(p\right)\right),$$

Here, *p* denotes the current pixel position, *k* is the relative position index within the convolution kernel, $w_{k}$ denotes the learnable convolution kernel weight, and $\Delta_{k}\left(p\right)$ represents the dynamic offset vector. The convolution forms along the x-axis ($DSConv_{x}$)$K_{i\pm c}$ and along the y-axis ($DSConv_{y}$)$K_{j\pm c}$ are respectively defined as follows:

(7) $$K_{i\pm c}=\left\{\begin{array}{cc} \left(x_{i+c},\;y_{i+c}\right) & =\left(x_{i}+c,\;y_{i}+\sum_{i}^{i+c}\Delta y_{t}\right),\\ \left(x_{i-c},\;y_{i-c}\right) & =\left(x_{i}-c,\;y_{i}+\sum_{i-c}^{i}\Delta y_{t}\right), \end{array}\right.$$

(8) $$K_{j\pm c}=\left\{\begin{array}{cc} \left(x_{j+c},\;y_{j+c}\right) & =\left(x_{j}+\sum_{j}^{j+c}\Delta x,\;y_{j}+c\right),\\ \left(x_{j-c},\;y_{j-c}\right) & =\left(x_{j}+\sum_{j-c}^{j}\Delta y,\;y_{j}-c\right), \end{array}\right.$$

**Figure 3:**
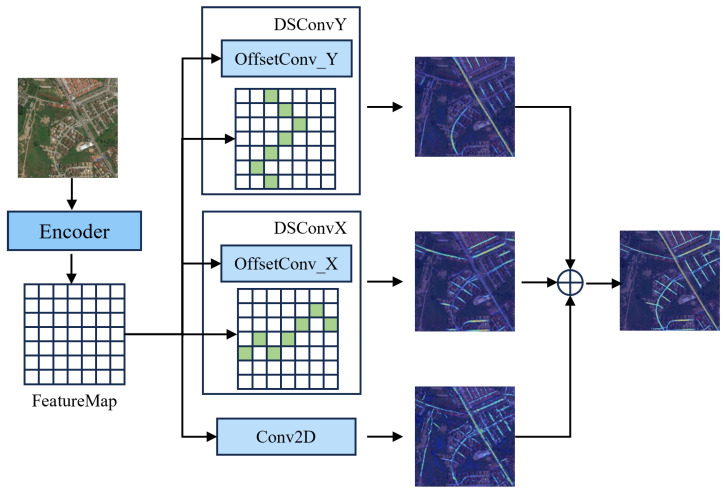
CI-Decoder: Enhancing connectivity features in the x and y directions improves the final decoding performance.

As shown in Figure 3, considering the spatially directionally distributed heterogeneity of road structures, we introduce three parallel convolutional branches for each layer: Standard 1×1 convolution: Preserves local semantic information and provides stable semantic context; Lateral serpentine convolution DSConvX: Serpentine convolution with dynamic offset sampling along the X-direction to capture lateral curvature structures; Longitudinal serpentine convolution DSConvY: Serpentine convolution with dynamic offset sampling along the Y-direction to enhance response to longitudinal bends. By introducing dynamic serpentine convolutions along the x and y axes, our decoder incorporates connectivity features across different directions, enabling consistent modeling of connectivity for curved roads.

The final feature representation after merging the three output streams is:

(9) $$F_{\mathrm{out}}=F^{\mathrm{std}}+F_{x}^{\mathrm{DSC}}+F_{y}^{\mathrm{DSC}}$$

This structure simulates the adaptive movement of the convolutional kernel along the curvature path of the road, enhancing the model’s ability to model winding, narrow, and obstructed roads.

Through this structure-aware perception enhancement mechanism, the model dynamically adjusts perception positions during inference to align with curved road paths, demonstrating superior topological preservation and detail recovery capabilities. Particularly in areas with occlusions, discontinuities, or small-scale bends, DSC significantly improves connectivity and edge coherence.

Experimental results also demonstrate that the decoder incorporating DSC achieves superior performance on evaluation metrics while maintaining or improving consistency in the general metric (mIoU), validating its effectiveness in modeling curvature.

### 3.4. Loss Function and Training Strategy

To simultaneously optimize pixel-level accuracy and the topological consistency of road structures, this paper proposes a joint loss function:

(10) $$L=\lambda_{1}\cdot L_{\mathrm{CE}}+\lambda_{2}\cdot L_{\mathrm{Topo}},$$

Among these, $L_{\mathrm{CE}}$ represents the standard cross-entropy loss, which optimizes pixel classification accuracy; $L_{\mathrm{Topo}}$ denotes the Topological Continuity Loss (TC-Loss), designed to enhance the model’s ability to capture the main structural features and connectivity relationships of roads. In our experiments, the weights $\lambda_{1}$ and $\lambda_{2}$ were set to 1.0.

The design of TC-Loss is based on the principles of Topological Data Analysis (TDA). First, TC-Loss can be viewed as a differentiable approximation of Persistent Homology. Persistent Homology studies the “birth-death” process of topological features at different scales in data, typically characterized by persistent diagrams. However, full persistent homology computation faces challenges of computational complexity and difficulty in end-to-end optimization within deep learning frameworks. Our method efficiently approximates the significance of topological features by detecting local maxima and minima in distance-transformed feature maps and defining “persistence” as the magnitude difference between these values.

**Figure 4:**
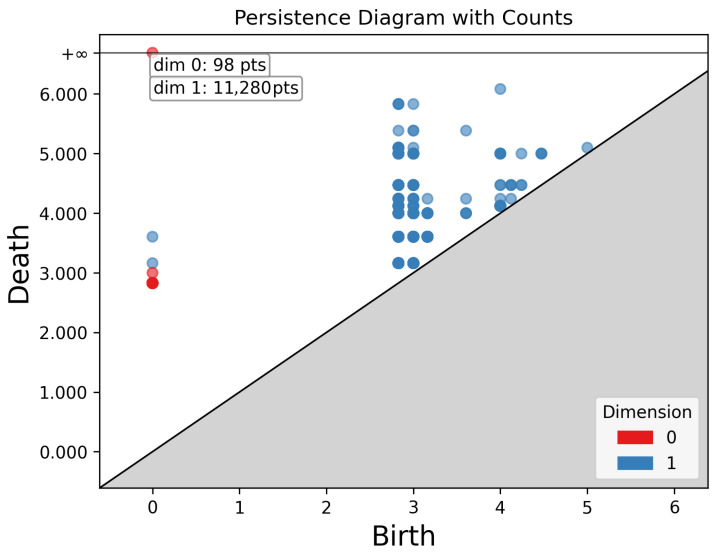
Persistence diagram: Enhancing connectivity features in the x and y directions improves the final decoding performance.

To gain a more intuitive understanding of persistence, Figure 4 illustrates the “birth-death” process of topological features such as connected components, cycles, and holes. The x-axis represents the “birth” value of a feature while the y-axis denotes its “death” value. Points above the diagonal line represent a topological feature, with the vertical distance from the point to the diagonal indicating the feature’s persistence or importance. Zero-dimensional features represent connected components. In road networks and scenes, numerous zero-dimensional points suggest roads may be fragmented or consist of multiple independent sections. The most persistent zero-dimensional points typically represent the primary road network. 1-dimensional features represent road loops or closed circuits. Their quantity reflects the complexity of the urban road network. High-persistence 1-dimensional features indicate significant large road loops.

To explicitly preserve the connectivity of road networks, we formulate the segmentation output as a topological space. Let (X,T) be a 2D pixel grid endowed with the adjacency-induced topology, and let Ω⊂X denote the foreground road region. From a topological perspective, the connectivity of Ω is characterized by its zero-dimensional homology group $H_{0}\left(\Omega \right)$, whose rank (i.e., Betti number $\beta_{0}$) counts the number of connected components. Unlike geometric or structural features that depend on local shapes, curvature, or gradients, topology concerns connectivity invariants independent of deformation. Thus, maintaining the stability of $H_{0}\left(\Omega \right)$ is crucial for preventing road breaks.

To obtain a differentiable approximation of persistent homology, we compute the Euclidean distance transform $d:\Omega \to R^{+}$, where d(x,y) gives the shortest distance to the boundary ∂Ω. This scalar field induces a Morse function whose critical points reflect topological characteristics: local maxima $M^{\max}$ correspond to centers of connected components, while local minima $M^{\min}$ lie near boundaries or potential disconnection points.

For each paired extremum $\left(m_{i}^{\max},m_{i}^{\min}\right)$, the persistence value

(11) $$\pi_{i}=d\left(m_{i}^{\max}\right)-d\left(m_{i}^{\min}\right)$$

approximates the birth–death lifetime of the corresponding topological feature in the 0-dimensional persistent homology diagram. We aggregate these values into a persistence field:

(12) $$P\left(x,y\right)=\sum_{i}\pi_{i}\;\delta \left(x-x_{i},\;y-y_{i}\right),$$

which highlights stable, long-range road structures.

To efficiently approximate this process within a neural network, we replace explicit persistence computation with multi-scale pooling (3×3, 5×5, 7×7 kernels) applied to the prediction and ground truth. This operation simulates the aggregation of road topology at different spatial scales, reinforcing long-range connectivity and suppressing small, noisy fragments.

Based on these representations, we define a topology-aware loss that aligns the extrema and persistence fields between prediction and ground truth:

(13) $$\begin{array}{cc} L_{\mathrm{Topo}}= & \mathrm{MSE}\left(M_{\mathrm{pred}}^{\max},M_{\mathrm{gt}}^{\max}\right)+\mathrm{MSE}\left(M_{\mathrm{pred}}^{\min},M_{\mathrm{gt}}^{\min}\right)\\ \; & \;+\mathrm{MSE}\left(P_{\mathrm{pred}},P_{\mathrm{gt}}\right). \end{array}$$

This loss provides a differentiable surrogate to persistent homology by enforcing consistency in (1) the location of critical points, and (2) the persistence magnitudes of connected components. It therefore complements geometric and structural losses by directly regulating the topological invariants of predicted road networks. By incorporating $L_{\mathrm{Topo}}$ into training, the network learns to preserve global road connectivity even under occlusion, curvature deformation, or multi-resolution appearance variations.

In summary, TC-Loss effectively captures connectivity changes in road trunks by aligning predictions with local extrema and persistence features of actual structures. This mitigates issues such as fracture, offset, and interruption prediction, further enhancing robustness in identifying long-distance narrow roads and tortuous structures. In terms of the number of parameters introduced by the new module, The MRFE introduces only lightweight depth-wise operations, contributing less than 3% additional parameters, while the CI-Decoder adds directional convolutions that increase computation marginally. TC-Loss is computed from downsampled distance maps, making its overhead negligible. Overall, TopoRF-Net delivers notable performance gains with only a small number of additional parameters, highlighting the efficiency of our design.

## 4. Experimental Setup and Results Analysis

### 4.1. Experimental Setup and DataSets

To evaluate the effectiveness of the proposed method, we conducted experiments on two widely-used road extraction benchmark datasets: the DeepGlobe Road dataset [2] and the Massachusetts Road dataset [60]. These datasets cover diverse rural and urban scenes, with variations in spatial resolution, object scales, and road network topology, providing a comprehensive testbed for robustness assessment.

The DeepGlobe Road dataset comprises high-resolution satellite imagery from multiple geographic regions, featuring complex environments such as rural landscapes, mountainous areas, and sparse road networks. Each image is annotated with pixel-level road labels. Following the official protocol, we split the dataset into 6226 training, 1240 validation, and 1217 test images.

The Massachusetts Road dataset consists of 1-m resolution aerial imagery covering urban and suburban areas of Massachusetts. It is particularly suitable for evaluating dense urban road networks extraction and exhibits strong structural consistency. The dataset includes 1108 images for training, 14 for validation, and 49 for testing.

All experiments were implemented in PyTorch 2.4.1 and performed on a workstation equipped with 4 NVIDIA RTX 3090 GPUs. During training, images were cropped into 512×512 patches with a batch size of 8 per GPU. We adopted the AdamW optimizer with an initial learning rate of 6 × 10^−5^, coupled with a cosine decay schedule and warm-up strategy.

### 4.2. Evaluation Indicators

In road extraction tasks, a comprehensive evaluation requires assessing both pixel-level classification accuracy and the spatial consistency between predicted and ground-truth regions. To this end, five commonly used indicators are adopted: Overall Accuracy (OA), Intersection over Union (IoU), Precision, Recall, and F1-score.

(1) Overall Accuracy (OA). OA measures the global classification correctness of all pixels in an image and is defined as:

(14) $$\mathrm{OA}=\frac{TP+TN}{TP+TN+FP+FN},$$

where TP and TN denote correctly predicted road and background pixels, respectively, while FP and FN represent incorrectly predicted and missed road pixels. Although OA reflects the overall reliability of classification, it can be influenced by foreground-background imbalance.

(2) Intersection over Union (IoU). IoU measures the spatial overlap between the predicted and reference road regions:

(15) $$\mathrm{IoU}=\frac{\left|P\cap G\right|}{\left|P\cup G\right|},$$

where *P* and *G* denote the sets of predicted and ground-truth road pixels, respectively. A higher IoU indicates better spatial alignment and boundary precision, which is particularly important for narrow or disconnected road structures.

(3) Precision and Recall. Precision evaluates the proportion of correctly predicted road pixels among all pixels predicted as roads, while Recall measures the proportion of correctly detected road pixels among all true road pixels:

(16) $$\mathrm{Precision}=\frac{TP}{TP+FP},\;\mathrm{Recall}=\frac{TP}{TP+FN}.$$

High Precision indicates fewer false positives while high Recall implies fewer missed detections. In road extraction, Recall is crucial for maintaining connectivity, whereas Precision ensures spatial reliability.

(4) F1-score. F1-score provides a balanced measure between Precision and Recall, defined as their harmonic mean:

(17) $$F1-\mathrm{score}=\frac{2\times \mathrm{Precision}\times \mathrm{Recall}}{\mathrm{Precision}+\mathrm{Recall}}.$$

This metric is sensitive to both false positives and false negatives, making it an effective indicator of structural completeness and overall segmentation robustness.

(5) Summary. IoU and F1-score emphasize regional and structural consistency, while OA and Precision–Recall focus on classification accuracy and detection reliability. By jointly analyzing these indicators, a comprehensive evaluation of both the pixel-level and topological performance of the road extraction model can be achieved.

### 4.3. Analysis of Experimental Results for the DeepGlobe Road Dataset

Results on the DeepGlobe-Road dataset are shown in Table 1. Despite all methods achieving high overall accuracy (OA > 97%), confirming reliable pixel-level classification, substantial disparities persist in road-specific performance. This is evident from the significant variations in IoU, F1-score, Precision, and Recall, highlighting distinct capabilities in extracting the target road class.

Traditional CNN architectures like UNet exhibit limited performance in IoU (61.66%) and F1-score (76.28%), with a recall rate of only 69.95%, indicating significant omissions at complex intersections and narrow road segments. DeepLabV3 achieves a larger receptive field by incorporating dilated convolutions, boosting IoU to 68.29% and F1-score to 81.16%. However, with a parameter size (Params) as large as 66M, DeepLabV3 faces a compromise in computational efficiency despite improved accuracy.

Transformer-based methods demonstrate superior performance due to their enhanced global modeling capabilities. SegFormer achieves an IoU of 69.47% and an F1-score of 81.98%, maintaining a balanced performance while keeping a relatively low Params (45 M). SegNeXt also achieves strong Recall (79.68%), though its overall accuracy falls slightly short of SegFormer. Conversely, D-LinkNet and RoadFormer demonstrate outstanding Precision (84.95% and 70.39%, respectively), but their lower Recall results in relatively weaker F1-scores and IoU.

The proposed TopoRF-Net achieves state-of-the-art or near-state-of-the-art performance across multiple key metrics. Specifically, it achieves an IoU of 69.76%, an F1-score of 82.18%, and Precision and Recall of 85.50% and 79.12%, respectively, outperforming most comparative methods. While maintaining high overall accuracy (OA = 98.57%) and a low Params (56 M), TopoRF-Net effectively balances detection precision with road network completeness. This advantage stems from the MRFE capturing multi-scale context, the dynamic snake convolution in the CI-Decoder modeling curvature for winding roads, and the Topological Constraint Loss (TC-Loss) optimizing topological connectivity. Consequently, TopoRF-Net significantly reduces road breaks and omissions in complex scenarios, better preserving the integrity of the overall road network.

The proposed TopoRF-Net method demonstrates superior overall performance compared to existing approaches, achieving 69.76% in road IoU and 79.16% in road pixel accuracy—both representing the best results among all compared methods. Simultaneously, the method maintains high performance on background categories, achieving an IoU of 98.52% and an Accuracy of 99.43%, demonstrating excellent foreground-background balance capability. Notably, TopoRF-Net features a parameter size of only 56 M, significantly lower than UPerNet (120 M) and D-LinkNet (218 M), fully demonstrating its superior balance between accuracy, connectivity, and efficiency. This validates the effectiveness of multi-receptive field enhancement and dynamic snake convolutions in enhancing road structure preservation while maintaining global classification accuracy.

### 4.4. Analysis of Experimental Results from the Massachusetts Dataset

Table 2 summarizes the experimental results on the Massachusetts dataset, where all metrics except overall accuracy (OA) are calculated for the road category. It can be observed that traditional CNN architectures (such as UNet and DeepLabV3) achieve over 96% overall accuracy but exhibit limited performance in IoU, F1-score, and recall, struggling to effectively preserve the slender structure of road objects. Taking UNet as an example, its IoU is 57.83%, and its F1-score is 73.28%. Although it achieves high accuracy, its recall rate is only 67.09%, indicating that it tends to omit some road pixels in complex scenes.

In contrast, Transformer-based methods such as UPerNet and SegFormer demonstrate improvements in both IoU and F1-score. For instance, UPerNet achieves an IoU of 59.54%, while SegFormer attains an F1-score of 74.02%, indicating that global context modeling positively impacts road extraction tasks. However, these approaches still struggle with precisely locating narrow road segments and complex intersection areas. On the other hand, D-LinkNet achieved the highest precision (85.13%), but its recall rate was only 60.36%, indicating that while it is sensitive to major road areas, it struggles to capture the complete road network.

The proposed TopoRF-Net achieves balanced and outstanding performance across multiple metrics. Specifically, it achieves an IoU of 59.68%, an F1-score of 74.75%, a Precision of 77.98%, Recall of 71.77%. It effectively balances precision and recall in road extraction while ensuring overall accuracy (96.65%) and parameter efficiency (56M). This is achieved through the introduced MRFE, which captures road texture features at different scales during the encoding phase; The CI-Decoder combined with dynamic snake convolutions aids in restoring continuity for winding and narrow road segments; additionally, the Topology Constraint Loss (TC-Loss) further reduces occurrences of discontinuities and structural gaps. Consequently, TopoRF-Net not only achieves overall accuracy comparable to state-of-the-art methods on the Massachusetts dataset but also demonstrates significant advantages in road connectivity and structural integrity.

The proposed TopoRF-Net method achieves state-of-the-art performance on the Massachusetts dataset. For road classification, it achieves an IoU of 59.68% and a pixel accuracy of 71.77%, significantly outperforming other methods. Simultaneously, it maintains high stability for background classification with an IoU of 96.48% and an accuracy of 98.50%. Considering model size, TopoRF-Net features only 56 M parameters—far fewer than UPerNet (120 M) and D-LinkNet (218 M). This demonstrates the proposed method’s advantage in balancing accuracy and road structural connectivity while maintaining lightweight efficiency. It fully validates the effectiveness of the topology-constrained loss and multi-receptive field feature enhancement module in improving road connectivity modeling.

### 4.5. Visualization Results Analysis

**Figure 5:**
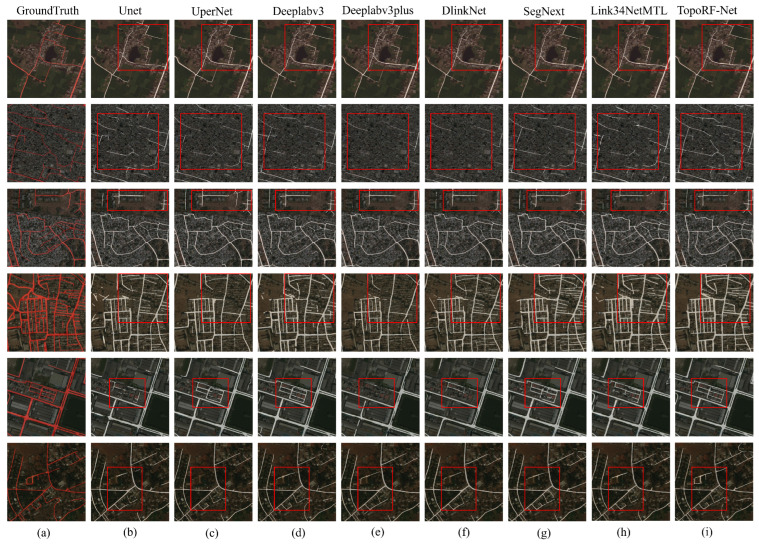
Visualization Results of the Deepglobe Dataset: Ground truth is overlaid on the orignal images in (**a**), followed by segmentations generated by (**b**) UNet, (**c**) UperNet, (**d**) Deeplabv3+, (**e**) DUSA-UNet, (**f**) DlinkNet, (**g**) SegNext, (**h**) LinkNet34MTL, and (**i**) TopoRFNet. Red squares are used to highlight representative regions where noticeable differences appear across methods, helping illustrate the visual advantages of our approach.

**Figure 6:**
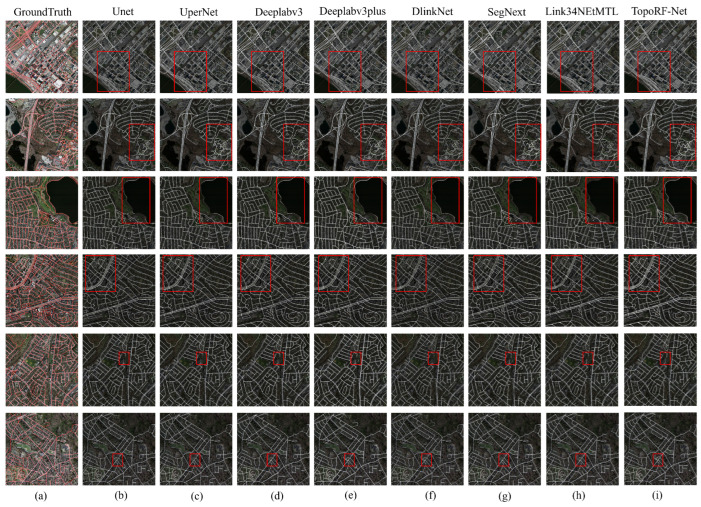
Visualization Results of Massachusetts Dataset: Ground truth is overlaid on the orignal images in (**a**), followed by segmentations generated by (**b**) UNet, (**c**) UperNet, (**d**) Deeplabv3+, (**e**) DUSA-UNet, (**f**) DlinkNet, (**g**) SegNext, (**h**) LinkNet34MTL, and (**i**) TopoRFNet. Red squares are used to highlight representative regions where noticeable differences appear across methods, helping illustrate the visual advantages of our approach.

Figure 5 and Figure 6 present visual comparisons between TopoRF-Net and state-of-the-art methods on the DeepGlobe and Massachusetts datasets. It can be observed that traditional methods such as UNet and DeepLabV3+ exhibit noticeable road discontinuities and boundary shifts in complex scenarios, particularly at intersections, narrow roads, and areas obscured by vegetation or buildings. Predictions often show discontinuities or even missing segments in these regions. In contrast, TopoRF-Net maintains high road connectivity and overall structural integrity in these challenging scenarios.

This performance improvement is closely tied to three key design elements proposed in this paper. First, the introduction of the MRFE module in the backbone network significantly enhances the encoder’s multi-scale feature perception capabilities. Through parallel multi-receptive field convolutions, MRFE effectively models road features at different scales, enabling the model to demonstrate robust performance when processing both narrow roads and wide highways. The Connectivity-Integrated Decoder (CI-Decoder) then perceives local curvature through dynamic serpentine convolutions during decoding. Combined with a global gating mechanism, it achieves adaptive fusion of cross-layer features. This design facilitates smoother road boundary reconstruction and effectively reduces discontinuities in intersection regions. Finally, the proposed Topological Consistency Loss (TC-Loss) further reinforces the model’s preference for main road trunks. This ensures prediction results maintain pixel-level accuracy while guaranteeing the consistency and stability of the global topological structure.

In summary, TopoRF-Net not only outperforms existing methods in pixel classification accuracy but also exhibits significant advantages in structural connectivity. Visualization results demonstrate how this approach can more accurately reconstruct the continuous skeleton of road networks in complex remote sensing scenarios, providing a more reliable foundation for subsequent traffic flow modeling and path planning.

### 4.6. Ablation Study

To validate the independent contributions of the key modules proposed in this paper to model performance, we conducted systematic ablation experiments on the DeepGlobe and Massachusetts datasets. The experiments examined the roles of the MRFE, CI-Decoder, and Topological Persistence Loss function (TC-Loss), respectively. Table 3 summarizes the relevant results.

Table 3 presents the results of stepwise ablation experiments conducted on the DeepGlobe and Massachusetts datasets. The findings demonstrate that each module contributes significantly to the model’s performance.

On the DeepGlobe dataset, the baseline model (MiT-B3 + CE) achieved an IoU-road (IoU metric for road segments) of 66.24% and an Acc-road (Acc metric for road segments) of 75.43%. After introducing the MRFE module, IoU-road increased to 68.15% (+1.91%) and Acc-road rose to 77.92% (+2.49%), demonstrating that multi-receptive field enhancement effectively improves the model’s perception capabilities for multi-scale roads. Further incorporating the CI-Decoder yields IoU-road and Acc-road of 68.71% and 78.57%, respectively, representing improvements of 0.56% and 0.65% over MRFE. This demonstrates the decoder’s advantage in preserving road connectivity through dynamic snake-based convolutions. Finally, incorporating TC-Loss elevated TopoRF-Net’s IoU-road to 69.76% and Acc-road to 79.16%, representing cumulative improvements of 3.52% and 3.73% over the baseline. This validates the effectiveness of topological persistence constraints in reducing road discontinuities and missing segments.

On the Massachusetts dataset, the baseline model achieved an IoU-road and Acc-road of 57.88% and 67.10%. After incorporating MRFE, both metrics improved to 58.86% and 69.07%, representing increases of 0.98% and 1.97%, respectively. This demonstrates that the module can enhance the recognition of narrow roads, even in low-resolution scenarios. When combined with CI-Decoder, IoU-road further went up to 59.54% and Acc-road to 70.21%, showing increases of 0.68% and 1.14% compared to using MRFE alone. Finally, incorporating TC-Loss resulted in TopoRF-Net achieving an IoU-road of 59.68% and Acc-road of 71.77%, representing overall improvements of 1.80% and 4.67% over the baseline. This demonstrates that structural loss plays a crucial role in maintaining the overall topological consistency of roads.

In summary, the MRFE module primarily enhances multi-scale perception capabilities, the CI-Decoder strengthens local curvature modeling and global fusion capabilities, while the TC-Loss constrains prediction results at the topological level. The synergistic interaction among these three components enables TopoRF-Net to achieve state-of-the-art performance on both datasets.

## 5. Discussion and Conclusions

This paper proposes TopoRF-Net, a structure-aware framework for road extraction tasks. It integrates a Multi-Receptive Field Enhancement module (MRFE) in the encoder, a Connectivity-Integrated Decoder (CI-Decoder) with dynamic snake convolutions in the decoder, and a Topology Persistence Constraint Loss (TC-Loss). To improve clarity and avoid redundant technical descriptions, we restructured this section to present the key contributions concisely. TopoRF-Net effectively addresses structural discontinuity and fragmentation in long, winding, or occluded roads. Experimental results demonstrate consistent improvements on both the DeepGlobe and Massachusetts datasets, showing gains in pixel-level accuracy as well as topological connectivity, thus validating the robustness and applicability of the proposed framework.

Limitations: Despite its strong performance, several limitations remain. First, although MRFE enhances multi-scale representation learning, its receptive field is still limited by convolutional kernel size, restricting ultra-long-range feature modeling. Second, the dynamic convolutions in CI-Decoder preserve road curvature effectively but may introduce additional computational cost on very large datasets. Third, the topology persistence loss, while effective for structural consistency, can be sensitive to noisy labels or severe annotation errors, potentially affecting optimization stability.

Future Work: Future research may extend this work in several directions. (1) Incorporating graph neural networks or implicit neural representations in the decoder could further strengthen global structural reasoning. (2) Exploring adaptive receptive-field expansion mechanisms may improve context coverage while maintaining lightweight computation. (3) Integrating multi-source data such as optical imagery and LiDAR could enhance generalization in complex urban or forest environments. (4) Developing more robust topology-aware constraints would improve performance under noise, weak supervision, or cross-domain transfer. Additionally, combining remote sensing indices or knowledge graph priors [66] may enrich semantic consistency and interpretability of extracted road networks.

Conclusion: In summary, TopoRF-Net demonstrates robust performance and strong topological awareness in road extraction tasks.

By simplifying structural components in this concluding section, we aim to highlight the framework’s practical benefits and its potential to inspire future research on connectivity-aware remote sensing segmentation.

## Figures and Tables

**Table 1:** Experimental Results for Each Method in the DeepGlobe-Road Dataset (All metrics except OA are single-class metrics for the road category).

Method	Params (M)	OA (%)	IoU (%)	F1-Score (%)	Precision (%)	Recall (%)
UNet [20]	29	98.18	61.66	76.28	83.88	69.95
UPerNet [61]	120	98.43	67.48	80.59	83.22	78.11
DeepLabV3 [6]	66	98.50	68.29	81.16	**85.58**	77.17
DeepLabV3+ [62]	41	98.04	58.04	73.45	84.81	64.77
SegFormer [4]	45	98.55	69.47	81.98	85.41	78.82
D-LinkNet [63]	218	98.05	58.09	73.49	84.95	64.75
RoadFormer [28]	59.2	97.21	59.63	75.60	70.39	**81.65**
UCTransNet [29]	66.2	97.10	57.46	73.99	71.67	76.48
SegNeXt [64]	49	98.41	67.61	80.68	81.70	79.68
DUSA-UNet [65]	66.9	97.53	67.77	81.34	83.50	79.29
LinkNet34MTL [9]	22	98.35	65.63	79.25	83.62	75.31
TopoRF-Net (Ours)	56	**98.57**	**69.76**	**82.18**	85.50	79.12

Note: Bold values indicate the best performance for each metric.

**Table 2:** Experimental Results for Each Method in the Massachusetts Dataset (All Metrics Except OA Are Single-Class Metrics for Road Categories).

Method	Params (M)	OA (%)	IoU (%)	F1-Score (%)	Precision (%)	Recall (%)
UNet [20]	29	96.62	57.83	73.28	80.73	67.09
UPerNet [61]	120	**96.74**	59.54	74.64	80.53	69.56
DeepLabV3 [6]	66	96.12	53.89	70.04	74.89	65.77
DeepLabV3+ [62]	41	96.49	59.67	74.74	74.31	**75.18**
SegFormer [4]	45	96.58	58.75	74.02	77.82	70.57
D-LinkNet [63]	218	96.54	54.60	70.64	**85.13**	60.36
RoadFormer [28]	59.2	96.02	53.82	64.65	62.07	67.47
UCTransNet [29]	66.2	96.18	57.87	71.63	74.24	69.21
SegNeXt [64]	49	96.07	54.03	70.16	73.59	67.03
DUSA-UNet [65]	66.9	96.06	58.61	71.62	74.49	68.98
LinkNet34MTL [9]	22	96.45	57.34	72.88	76.94	69.23
TopoRF-Net (Ours)	56	96.65	**59.68**	**74.75**	77.98	71.77

Note: Bold values indicate the best performance for each metric.

**Table 3:** Ablation Study of TopoRF-Net Components on the DeepGlobe and Massachusetts Datasets (Road Metrics).

Configuration	DeepGlobe		Massachusetts	
	IoU (%)	Accuracy (%)	IoU (%)	Accuracy (%)
Baseline (MiT-B3 + CE)	66.24	75.43	57.88	67.10
+MRFE	68.15	77.92	58.86	69.07
+MRFE + CI-Decoder	68.71	78.57	59.54	70.21
+MRFE + CI-Decoder + TC-Loss				
(TopoRF-Net)	69.76	79.16	59.68	71.77

Results show road extraction performance metrics only. Background metrics are omitted for clarity.

## Data Availability

The data supporting the findings of this study are openly available in a public repository. The Massachusetts Road dataset is available at https://www.cs.toronto.edu/~vmnih/data/, accessed on 24 November 2025. The DeepGlobe Road dataset can be found at https://competitions.codalab.org/competitions/18467, also accessed on 24 November 2025.
